# Medical Women and the Royal Free Hospital

**Published:** 1891-03-28

**Authors:** Elizabeth Garrett Anderson

**Affiliations:** 4, Upper Berkeley Street


### EDITOR'S LETTER-BOX.

[OUR Correspondents are reminded that prolixity is a great bar to publication, and that brevity of style and conciseness of statement greatly facilitate early insertion.]

### MEDICAL WOMEN AND THE ROYAL FREE HOSPITAL.

SIR,—My attention has been directed to an article in your issue of the 14th inst., in which comments are made on the evidence given by me before the House of Lords Committee upon Hospitals, and in which the action of the Royal Free Hospital is somewhat unfavourably criticised. Without entering upon any discussion as to the general tenour of the friendly arguments brought forward in your article, I wish to correct two mistakes it contains as to matters of fact. It is not correct to say that the action of the Royal Free Hospital in admitting female medical students to the wards of the hospital was an almost worthless concession because the students have to go elsewhere for their practical midwifery. It is also incorrect to say that the concession was made under the pressure of public opinion. The admission of the students of the London School of Medicine for Women to the wards of the Royal Free Hospital has been for many years—and is still—of the greatest advantage to them, and the privilege of this admission was given at a time when public opinion had put no pressure at all upon the authorities of the hospital, and when it was at least possible that by their liberal action to the female medical students some part of the public support hitherto given to the Royal Free Hospital might have been alienated. This has happily not proved to be the case, but the authorities of the hospital certainly acted in advance of public opinion instead of in obedience to it. The promoters of the medical education of women can never have anything but a keen and hearty appreciation of the valuable help rendered to their cause by the Royal Free Hospital.—I am, sir, yours,

ELIZABETH GARRETT ANDERSON, M.D.

4, Upper Berkeley Street,
March 20th, 1891.

